# Impaired long-term depression mediated by group I metabotropic glutamate receptors, but not muscarinic acetylcholine receptors in juvenile-onset diabetes rats

**DOI:** 10.1016/j.ibneur.2026.08.015

**Published:** 2026-08-17

**Authors:** Hayuma Otsuka, Sachie Sasaki-Hamada, Hitoshi Ishibashi, Jun-Ichiro Oka

**Affiliations:** a Laboratory of Pharmacology, Faculty of Pharmaceutical Sciences, Tokyo University of Science, Chiba 278-8510, Japan; b Department of Physiology, School of Allied Health Sciences, Kitasato University, Kanagawa 252-0329, Japan

**Keywords:** LTD, CA1, Diabetes, mGluR, mAChR

## Abstract

Type 1 diabetes mellitus is a chronic disease where the destruction of insulin-producing pancreatic β cells causes hyperglycemia and its onset typically occurs during childhood or adolescence. We previously reported that long-term depression (LTD) in the CA1 region of the hippocampus, induced by a low-frequency stimulation to the Schaffer collaterals, was attenuated in juvenile-onset diabetes (JDM) rats. We herein investigated whether LTD was triggered by the activation of G-protein coupled group I metabotropic glutamate receptors (mGluR-LTD) or M1 muscarinic acetylcholine receptors (mAChR-LTD) at Schaffer collateral-CA1 synapses in hippocampal slices from JDM rats. Our results indicated that the magnitude of mGluR-LTD induced by the group I mGluRs agonist (S)-3,5-dihydroxyphenylglycine (DHPG) was smaller in JDM rats than in age-matched control rats. Paired-pulse facilitation (PPF) is primarily associated with enhanced presynaptic transmitter release. To investigate the interaction between PPF and mGluR-LTD, PPF ratios conducted at interstimulus intervals (ISIs) of 50 and 200 ms were compared at the baseline and LTD stage. In control rats, the PPF ratio at ISIs of 50 ms was higher during the mGluR-LTD stage than at the baseline. However, PPF ratios at ISIs of 50 and 200 ms were higher during the mGluR-LTD stage than at the baseline in JDM rats. In contrast, the magnitude of mAChR-LTD did not significantly differ between control and JDM rats. Therefore, the expression of mGluR-LTD in JDM rats may be associated with presynaptic changes, since the increase in the PPF ratio occurred concomitantly with the attenuation of LTD.

## Highlights

• mGluR-LTD at hippocampal CA3-CA1 synapses was impaired in JDM rats.
• mGluR-LTD in JDM rats did not require the postsynaptic activation of NMDA receptors.
• mGluR-LTD in JDM rats was affected with presynaptic function.
• mAChR-LTD at hippocampal CA3-CA1 synapses was intact in JDM rats.

## 1 Introduction

Type 1 diabetes mellitus (T1DM) is a chronic disease where the destruction of insulin-producing pancreatic β cells causes hyperglycemia and its onset typically occurs during childhood or adolescence. The International Diabetes Federation estimated that 9.2 million people are living with T1DM, including 1.8 million younger than 20 years (International Diabetes Federation, 2025). A meta-analysis showed a higher incidence in the 5–9 and 10–14 age groups than in the 0–4 and 15–19 age groups (Hormazábal-Aguayo et al., 2024). Childhood-onset T1DM has been associated with cognitive deficits in intelligence, memory, attention, and executive functions (Ferguson et al., 2005, Geddes et al., 2008, McAulay et al., 2006).

We previously reported learning impairments and hippocampal long-term depression (LTD) deficits induced by low-frequency synaptic activity (LFS-LTD), but not long-term potentiation (LTP) by high-frequency synaptic activity, in juvenile-onset DM (JDM) rats (Iwai et al., 2009, Sacai et al., 2014, Sasaki-Hamada et al., 2012). Hebbian-type synaptic plasticity, including LTP and LTD, is considered to be the cellular basis of learning and memory formation in the brain (Diering and Huganir, 2018, Malenka and Bear, 2004). At CA3-CA1 synapses in the hippocampus, G_q/11_ coupled group I metabotropic glutamate receptors (mGluRs) and M1 muscarinic acetylcholine receptors (mAChRs) are expressed in pyramidal neurons, and have been implicated in the mechanisms underlying LTD (Hasselmo, 1999, Malenka and Bear, 2004, Oliet et al., 1997). The stimulation of group I mGluRs by perfusing the selective agonist DHPG induced a long-lasting decrease in synaptic activity (mGluR-LTD) (Palmer et al., 1997, Volk et al., 2007). Similarly, the stimulation of M1 mAChRs by the agonist McN-A−343 caused a long-lasting decrease in synaptic activity (mAChR-LTD) (Scheiderer et al., 2006). Since LTDs induced by these agonists diverge many mechanistic aspects from LFS-LTD (Collingridge et al., 2010, Warburton et al., 2003), we investigated mGluR-LTD and mAChR-LTD at Schaffer collateral-CA1 (SC-CA1) synapses in hippocampal slices from JDM rats. We also examined the mechanisms by which glutamate release and glutamate receptors affected the induction of mGluR-LTD in JDM rats.

## 2 Materials and methods

### 2.1 Ethics statement

All animal experiments were performed in accordance with the ARRIVE guidelines (https://www.nc3rs.org.uk/arrive-guidelines) and the guidelines of the National Institute of Health and Japan Neuroscience Society, and were approved by the Institutional Animal Care and Use Committee of Tokyo University of Science and Kitasato University.

### 2.2 Chemicals

4-[[[(3-Chlorophenyl)amino]carbonyl]oxy]-*N*,*N*,*N*-trimethyl−2-butyn−1-aminium chloride (McN-A−343), D−2-amino−5-phosphonopentanoic acid (D-AP5), and (RS)−3,5-dihydroxyphenylglycine (DHPG) were obtained from Tocris Cookson (Bristol, UK). STZ and carbamoylcholine chloride (CCh) were purchased from Sigma-Aldrich (St. Louis, MO, USA). All other chemicals were supplied by FUJIFILM Wako Pure Chemical Industries (Osaka, Japan).

### 2.3 Animals

We used male and female Wistar rats (Sankyo Labo Service Corporation and Nihon SLC, Shizuoka, Japan), and made every effort to minimize the number of animals used as well as pain and distress. JDM was induced by STZ (85 mg/kg, i.p.) in 17-day-old rats (Sacai et al., 2014, Sasaki-Hamada et al., 2012). STZ was dissolved in phosphate-buffered saline (PBS). Age-matched and PBS-treated rats were used as controls. To confirm the induction of DM, we measured glucose levels in venous blood samples using a glucose test meter (GUNZE Co., Kyoto, Japan or Nipro Co., Osaka, Japan) before experiments. A total of 21 JDM rats (22–24 weeks old), including 13 males and 8 females, were used in this study. At the time of the experiment, body weight and blood glucose levels of JDM rats were 177.5 ± 11.2 g and 622.3 ± 36.1 mg/dl (N = 21; N = number of animals). Animals were considered to be diabetic when glucose levels were higher than 300 mg/dl. In this study, 14 STZ-treated rats did not develop diabetes. All animals were kept in a controlled environment, with a 12–12-h light schedule, temperature of 23 ± 1ºC, and relative humidity of 55 ± 5%, and were provided *ad libitum* access to food and water.

### 2.4 Slice preparation

Hippocampal slices were prepared from 22- to 24-week-old rats. Rats were anesthetized with isoflurane and decapitated. Using a vibratome (DTK−1000, Dosaka, Kyoto, Japan), 300-μm-thick transverse hippocampal slices were cut in ice-cold dissection buffer (in mM; 234 sucrose, 2.5 KCl, 1.25 NaH_2_PO_4_, 0.5 CaCl_2_, 10 MgSO_4_, 26 NaHCO_3_, and 11 D-glucose) saturated with 95% O_2_ and 5% CO_2_. To avoid the possibility that DHPG, CCh, or McN-A−343 may change neuronal activity in the CA3 region (Huber et al., 2000, Scheiderer et al., 2006), we used hippocampal slices in which the CA3 region was removed immediately after cutting. Slices were recovered at 36ºC for 50 min in normal artificial CSF (ACSF) (in mM; 124 NaCl, 3 KCl, 2.5 CaCl_2_, 1.3 MgSO_4_, 1.24 KH_2_PO_4_, 10 D-glucose, and 26 NaHCO_3_).

### 2.5 Extracellular field potential recordings

Slices were maintained during recordings by being perfused at 2–3 ml/min with ACSF. Extracellular field EPSPs (fEPSPs) from the stratum radiatum of the hippocampal CA1 region were recorded using glass micropipettes (4–7 MΩ) with ACSF. Test stimuli (duration of 80 μs) were delivered once every 60 s using a bipolar tungsten electrode (pole separation of 150 μm; WPI, Sarasona, FL, USA), which was placed on the afferent fiber of the stratum radiatum in the CA1 region of the hippocampus. An evoked fEPSP was generated by an electric stimulator (SEM−3301; Nihon Koden, Tokyo, Japan) and isolator (SS−202j; Nihon Koden), filtered at 5 kHz, digitized at 20 kHz, and analyzed on a personal computer using a PowerLab/4 s system (AD Instruments, Castle Hill, Australia). Therefore, the amplitudes of evoked fEPSPs were measured in real time. To induce LTD, the stimulation intensity was set to elicit 60–70% of the maximal fEPSP amplitude. Chemical LTD was elicited by the application of 50 μM DHPG, 50 μM CCh, or 50 μM McN-A−343 for 10 min (Hou et al., 2006, Scheiderer et al., 2006).

To measure the paired-pulse ratio, a paired pulse stimulation was delivered at different inter-stimulus intervals (ISIs) during the baseline period and 70 min after the application of DHPG. ISIs between successive pulses were set as 50 and 200 ms. The paired-pulse ratio was analyzed offline using Igor Pro (WaveMetrics, OR, USA) after completion of the experiments. The ratio of the maximum negative slope of the second pulse to the first pulse was computed as the paired pulse ratio. The slope was measured by 30–80% of the fEPSP peak amplitude.

### 2.6 Western blot analysis

Whole hippocampi were dissected from control and JDM rats and then homogenized in lysis buffer with a protease inhibitor cocktail (P8340, Sigma-Aldrich). Protein concentrations were measured using a BCA colorimetric protein assay (Thermo Fisher Scientific Inc., Rockford, IL, USA), and 20 μg of purified protein was resolved by SDS-PAGE and transferred onto a PVDF membrane. Regarding antibody detection, after an incubation with 5% non-fat dried milk at room temperature for 1 h, membranes were incubated with the primary antibody anti-Homer1 (dilution 1:1000, GTX103278, Gene Tex, Irvine, CA, USA), anti-Homer1b/c (dilution 1:200, sc−25271, Santa Cruz Biotechnology, CA, USA), or anti-β-actin (dilution 1:5000, A5441, Sigma-Aldrich) at 4°C overnight. Immunoreactive bands were then probed with an HRP-conjugated secondary antibody for 1 h, developed using the ECL detection system (Cytiva, Uppsala, Sweden), and visualized with FUSION SOLO 7S Edge (Vilber, Collégien, France). The membranes were converted to digital images and protein expression was quantified by a densitometric analysis of each immunoreactive band using the Fusion-No system (version18.06-dSN; Vilber).

### 2.7 Data and statistical analyses

Data analyses were performed using Igor Pro (WaveMetrics). Data are shown as means ± S.E.M. Statistical analyses were conducted using GraphPad Prism 11 (Graphed software, San Diego, CA, USA) with the significance level set as *p* < 0.05. The sample size was determined based on previous studies (Otsuka et al., 2024, Otsuka et al., 2026). Data were checked for normality using the Shapiro-Wilk test. The Student’s *t-*test was used for normally distributed data. Non-normally distributed data were analyzed using the Mann-Whitney *U* test. An evaluation of PPF, based on comparisons between the second- and first-evoked potentials, was performed using the Wilcoxon matched-pairs signed-rank test.

## 3 Results

### 3.1 Impaired mGluR-LTD in JDM rats

**Fig. 1:**
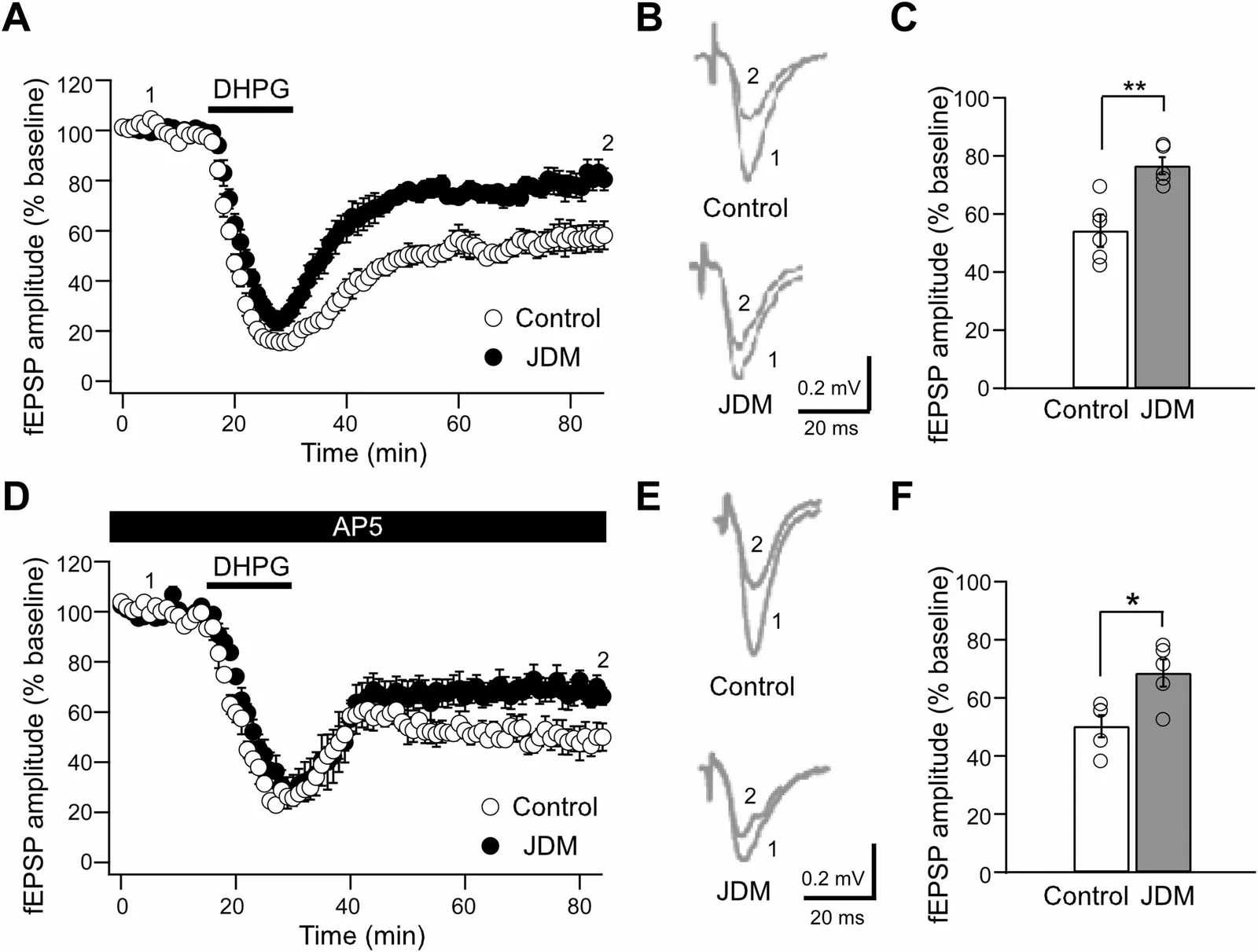
**Impaired DHPG-LTD at SC-CA1 synapses in JDM rats.** Hippocampal slices were obtained from control and JDM rats at 22–24 weeks. **(A)** The bath application of the group I mGluRs agonist, DHPG (50 μM, 10 min) to acute hippocampal slices induced LTD in control rats, and DHPG-LTD was attenuated in JDM rats. **(B)** Representative fEPSP traces at the time points indicated are shown for control (above) and JDM rats (bottom). **(C)** The bar graph shows the normalized fEPSP amplitude during the last 30 min of LTD induction, and the magnitude of DHPG-LTD was significantly smaller in JDM rats than in age-matched control rats (n = 6 in control, n = 5 in JDM; **p < 0.01) (N = 3−4, respectively). (**D**) DHPG-LT**D** was not affected by the NMDA receptor antagonist, D-AP5 (50 μM), in control or JDM rats (n = 5, respectively). (**E**) Representative fEPSP traces at the time points indicated are shown in control (above) and JDM rats (bottom). **(F)** The bar graph shows the normalized fEPSP amplitude during the last 30 min of LTD induction (n = 5 in control, n = 5 in JDM; *p < 0.05) (N = 3–4, respectively). Data are presented as the mean (± S.E.M.), and the significance of differences was set as *p* < 0.05. N = the number of animals, n = the number of slices.

We recorded fEPSPs at SC-CA1 synapses in acute hippocampal slices obtained from JDM and age-matched control rats. The average fEPSP amplitude during the 15 min before pharmacological activation was defined as 100%. We induced mGluR-LTD at hippocampal SC-CA1 synapses chemically with the group Ι mGluR agonist, DHPG (50 μM for 10 min). The application of DHPG elicited the transient depression of synaptic transmission, which was followed by a long-lasting depression (DHPG-LTD), in control and JDM rats (Fig. 1A, B). The reduction in the fEPSP amplitude was sustained for 70 min after the washout of DHPG in both groups; however, the magnitude of DHPG-LTD during the last 30 min was significantly attenuated in JDM rats (control fEPSP: 54.2 ± 5.6% of the baseline, n = 6; STZ fEPSP: 76.6 ± 3.0% of the baseline, n = 5, *p* = 0.002, the Student’s *t*-test) (Fig. 1C).

### 3.2 Effects of an NMDA receptor antagonist on mGluR-LTD

Since previous studies reported that DHPG enhanced NMDA receptor function (Fitzjohn et al., 1996), we investigated whether mGluR-LTD in JDM rats required the postsynaptic activation of NMDA receptors. Therefore, D-AP5, an NMDA receptor antagonist, was continuously perfused. In the presence of D-AP5, the reduction in the fEPSP amplitude was sustained for 70 min after the washout of DHPG in both control and JDM rats (Fig. 1D, E). Additionally, the magnitude of DHPG-LTD during the last 30 min was significantly attenuated in JDM rats (control fEPSP: 50.2 ± 3.8% of the baseline, n = 5; STZ fEPSP: 68.5 ± 4.7% of the baseline, n = 5, *p* = 0.016, the Student’s *t*-test) (Fig. 1F). These results suggest that the maintenance of DHPG-LTD was resistant to the activation of NMDA receptors in both control and JDM rats.

### 3.3 Expression of hippocampal Homer1 and Homer1b/c in JDM rats

**Fig. 2:**
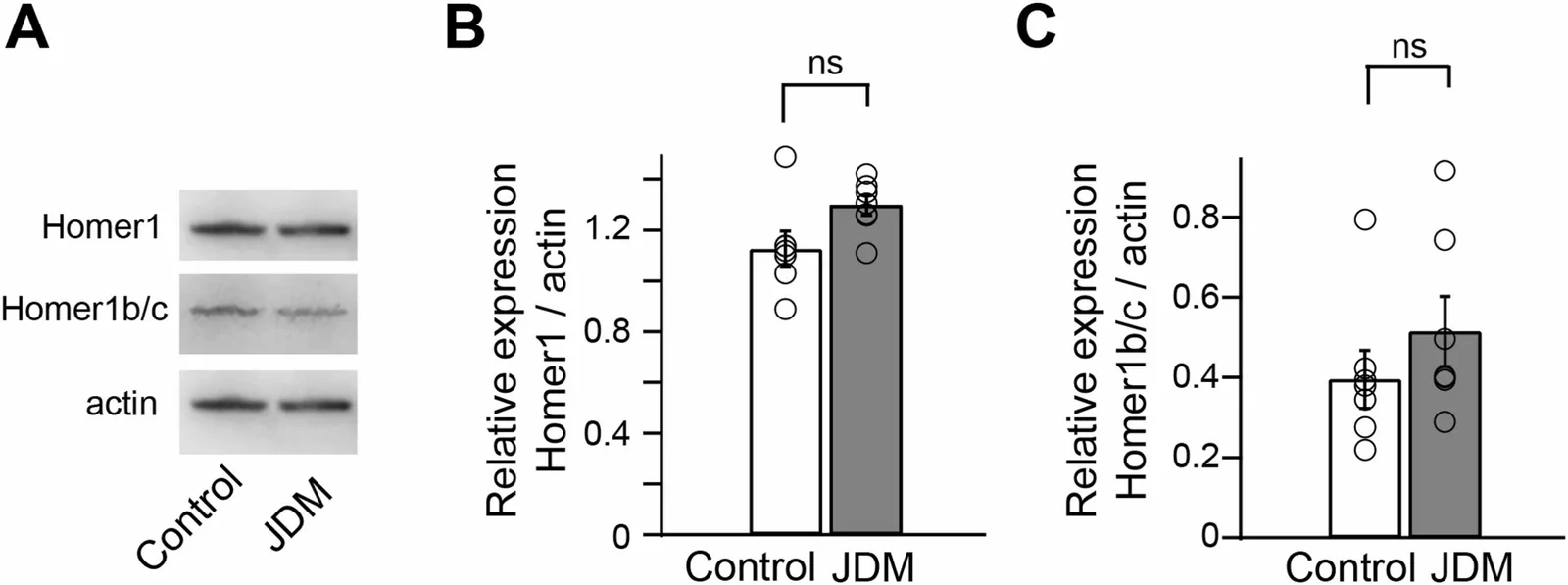
**Hippocampal Homer1 expression in JDM rats.** (**A**) Representative Western blots show Homer1 and Homer1b/c protein expression in the hippocampus of control and JDM rats. (**B, C**) The bar graph shows Homer1 and Homer1b/c immunoreactivities normalized to actin immunoreactivity. No significant differences were observed in Homer1 or Homer1b/c expression between control and JDM rats (n = 7, respectively, *p* > 0.05) (N = 7, respectively). Data are presented as the mean (± S.E.M.), and the significance of differences was set as *p* < 0.05. ns, not significant.

Homer1 proteins, which include Homer1b/c, are postsynaptic scaffolding proteins that bind to the intracellular tail of group I mGluRs and are an integral component of the postsynaptic mGluRs signaling complex (Brakeman et al., 1997, Kammermeier, 2008, Tu et al., 1998). We examined the expression levels of Homer1 and Homer1b/c in the hippocampus of JDM rats. The results of a Western blot analysis showed no significant differences in Homer1 or Homer1b/c protein expression between control and JDM rats (n = 7 respectively, *p* > 0.05, the Student’s *t*-test) (Fig. 2).

### 3.4 mGluR-LTD in JDM rats was affected by presynaptic function

**Fig. 3:**
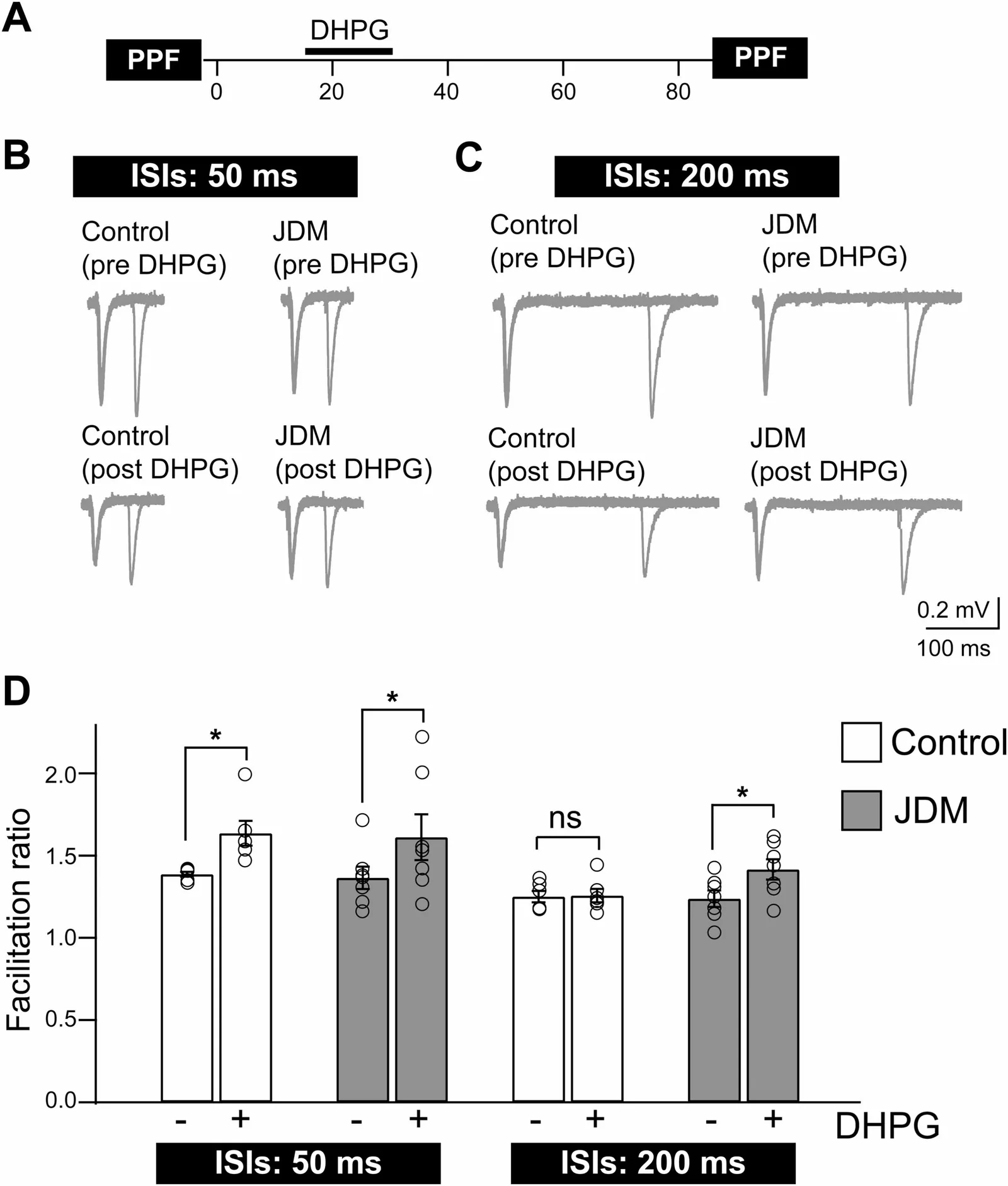
**Changes in the PPF ratio associated with DHPG-LTD in JDM rats.**Fig. 3A shows the time course used for PPF measurements. Two inter-stimulus paired pulse stimulations (50 and 200 ms) were delivered in the baseline period (pre DHPG) and 70 min after the application of DHPG (post DHPG). (**B, C**) Representative fEPSP traces in response to two consecutive stimulations are shown in control and JDM rats. (**D**) DHPG induced a significant increase in the PPF ratio with ISIs of 50 ms in control (n = 6; *p < 0.05) and JDM rats (n = 7; *p < 0.05). However, at ISIs of 200 ms, the application of DHPG did not affect the PPF ratio in control rats (n = 6; *p* > 0.05), while the PPF ratio increased in JDM rats (n = 7; *p < 0.05) (N = 3–4, respectively). Data are presented as the mean (± S.E.M.), and the significance of differences was set as *p* < 0.05. ns, not significant.

We compared PPF ratios between the baseline (pre DHPG) and LTD stage (post DHPG) to establish whether presynaptic mechanisms were involved in mGluR-LTD in control and JDM rats. PPF has been attributed to an increase in the amount of neurotransmitter released in response to a second stimulus (Katz and Miledi, 1968, Zucker, 1989). PPF was initially measured before and after LTD induction in the experiments shown in Fig. 3A. Additionally, the PPF analysis was performed in a separate set of experiments using a different cohort of animals from those used for the LTD recordings shown in Fig. 1. Statistical comparisons of the PPF ratio between pre DHPG and post DHPG were conducted with the Wilcoxon matched-pairs signed-rank test. In control rats, we found that the PPF ratio significantly increased after DHPG application at inter-stimulus intervals (ISIs) of 50, 80, and 120 ms (*p* < 0.05), but not at 200 ms (*p* = 0.6875). A previous study also showed that DHPG increased the PPF ratio at ISIs ranging from 20 to 150 ms (Faas et al., 2002). Therefore, we selected the ISIs of 200 ms, which is not affected by DHPG, whereas the ISIs of 50 ms was used as a DHPG-sensitive condition. In control rats, DHPG significantly increased the PPF ratio at ISIs of 50 ms (*p* = 0.0312), but not that at ISIs of 200 ms (n = 6, *p* = 0.6875) (Fig. 3B, C). However, the PPF ratio at both ISIs in JDM rats significantly increased after the application of DHPG (n = 7, *p* < 0.05) (Fig. 3D).

### 3.5 Cholinergic receptor-dependent LTD in JDM rats

**Fig. 4:**
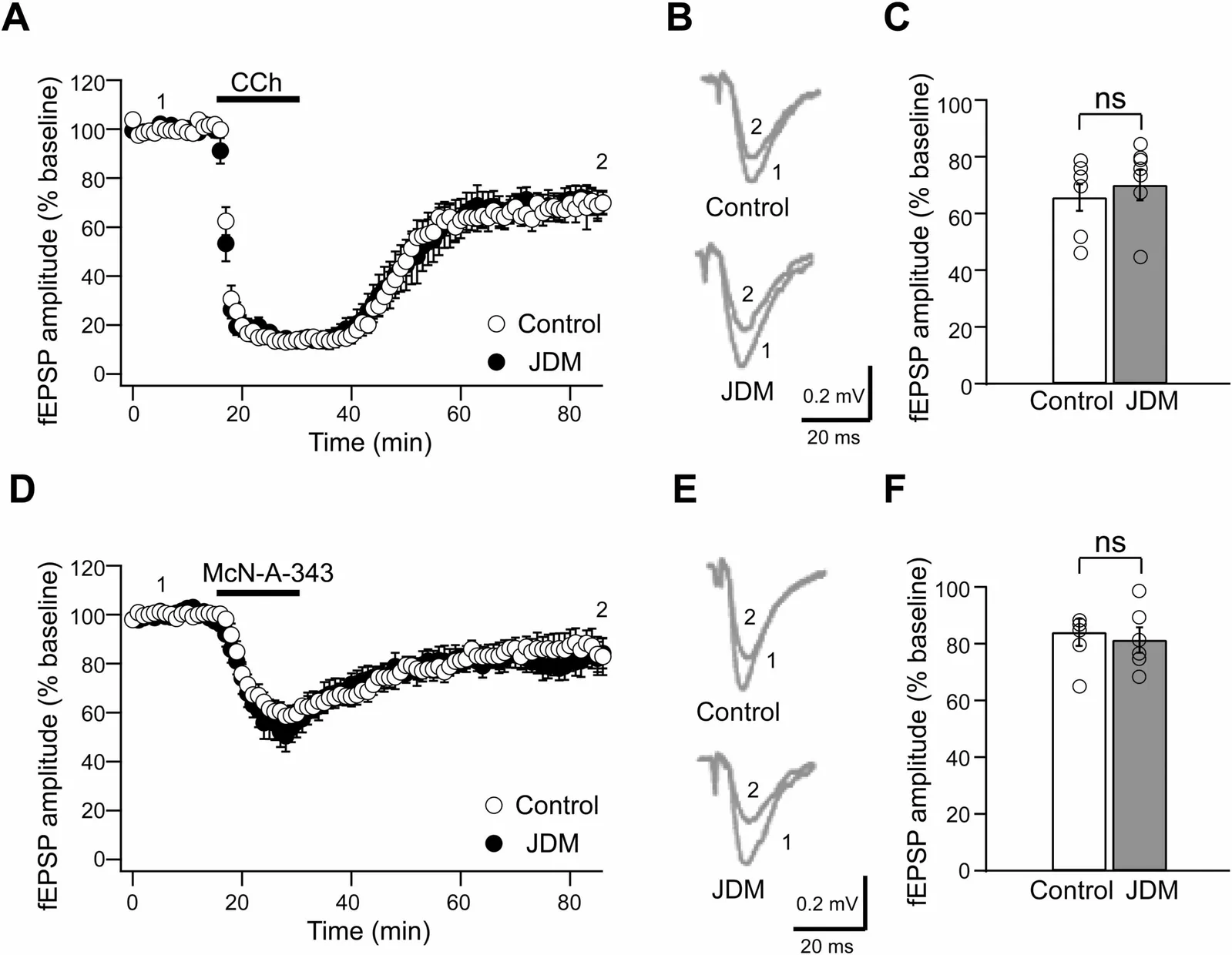
**mAChR-LTD at SC-CA1 synapses in JDM rats. (A)** The bath application of the non-selective cholinergic receptor agonist, CCh (50 μM, 10 min) to acute hippocampal slices induced LTD in both control (n = 7) and JDM rats (n = 6). **(B)** Representative fEPSP traces at the time points indicated are shown for control (above) and JDM rats (bottom). **(C)** The bar graph shows the normalized fEPSP amplitude during the last 30 min of LTD induction (n = 7 in control, n = 6 in JDM; *p* > 0.05) (N = 3–4, respectively). **(D)** The bath application of the selective muscarinic M1 receptor agonist, McN-A−343 (50 μM, 10 min) to acute hippocampal slices induced LTD in both control (n = 6) and JDM rats (n = 6). **(E)** Representative fEPSP traces at the time points indicated are shown for control (above) and JDM rats (bottom). **(F)** The bar graph shows the normalized fEPSP amplitude during the last 30 min of LTD induction (n = 6 in control, n = 6 in JDM; *p* > 0.05) (N = 3–4, respectively). Data are presented as the mean (± S.E.M.), and the significance of differences was set as *p* < 0.05. ns, not significant.

The application of the non-selective cholinergic receptor agonist, carbachol (CCh, 50 μM for 10 min) induced LTD at hippocampal SC-CA1 synapses. The application of CCh elicited the transient depression of synaptic transmission, which was followed by a long-lasting depression (CCh-LTD), in control and JDM rats (Fig. 4A, B). Fig. 4C shows that CCh-LTD was similarly induced in control and JDM rats (control fEPSP: 65.6 ± 4.7% of the baseline, n = 7; STZ fEPSP: 70.0 ± 5.4% of the baseline, n = 6, *p* > 0.999, the Mann-Whitney *U* test).

To confirm that CCh-LTD at hippocampal SC-CA1 synapses requires the activation of mAChRs, we used the selective muscarinic M1 receptor agonist, McN-A−343 (50 μM). We compared mAChR-LTD between control and JDM rats (Fig. 4D, E). Fig. 4F shows that mAChR-LTD was similarly induced in control and JDM rats (control fEPSP: 84.0 ± 4.8% of the baseline, n = 6; STZ fEPSP: 81.3 ± 4.5% of the baseline, n = 6, *p* = 0.688, the Student’s *t*-test).

## 4 Discussion

Group I mGluR subtypes (mGluR1 and mGluR5) and M1 mAChRs couple to the G_q/11_ protein and stimulate phospholipase C (PLC) to catalyze the formation of inositol phosphates and diacylglycerol, which results in the release of intracellular Ca²⁺ from stores via inositol triphosphate receptors (Bordi and Ugolini, 1999, Caulfield and Birdsall, 1998). Although group I mGluRs and M1 mAChRs activate the same G-protein coupled receptors and isoforms of PLC, different molecular mechanisms of LTD have been reported (Bodzęta et al., 2021, Dickinson et al., 2009). We previously demonstrated impairments in both mGluR-LTD and mAChR-LTD in young adult-onset STZ rats (Otsuka et al., 2024, Otsuka et al., 2026). In the present study, we showed impaired mGluR-LTD, but not mAChR-LTD in JDM rats. These results indicate that the effects of STZ-induced diabetes on LTD mediated by two different Gq-coupled receptors were dependent on age at disease onset.

Fig. 1 shows DHPG-LTD at CA3-CA1 synapses in hippocampal slices from control rats, while DHPG-LTD in JDM rats was attenuated. Previous studies demonstrated that mGluR5 antagonists blocked DHPG-LTD, but not mGluR1 antagonists (Fitzjohn et al., 1999, Huang and Hsu, 2006). Moreover, group I mGluR subtypes are distributed differently in the hippocampal CA1 and CA3 regions. Although mGluR5 is the most abundant in the CA1 region, mGluR5 and mGluR1 have both been found in the CA3 region (Berthele et al., 1998, Luján et al., 1996). A previous study on gene expression showed that hippocampal mGluR5 mRNA was significantly decreased in 7- and 90-week-old STZ-induced diabetic rats (Balakrishnan et al., 2009). mGluR5 endocytosis and intracellular sorting are important mechanisms for fine-tuning mGluR5 signaling and preventing receptor overstimulation. Since mGluR5-interacting proteins including CaMKIIα, Caveolin−1 and Norbin have been reported to be involved in the regulation of mGluR-LTD (Kalinowska and Francesconi, 2016), elucidating whether alterations in these molecules contribute to the impaired DHPG-LTD at CA3-CA1 synapses in hippocampal slices from 22- to 24-week-old JDM rats will be an important subject for future investigation.

DHPG may be permitting sufficient NMDA receptor activation during a basal synaptic stimulation to trigger LTD because DHPG was shown to potentiate the effects of NMDA receptor activation (Fitzjohn et al., 1996). However, the NMDA receptor antagonist AP5 did not affect DHPG-LTD at CA3-CA1 synapses in 20- to 32-week-old rats (Kumar and Foster, 2007). In the present study, we also observed DHPG-LTD in the presence of AP5 in both control and JDM rats (Fig. 1D), which suggests that NMDA activation was not required for DHPG-LTD in JDM rats.

The postsynaptic scaffold protein Homer1b/c was previously shown to be necessary for the expression of DHPG-LTD (Gimse et al., 2018). Additionally, the interaction of mGluR5 with Homer was required for the ability of the receptor to induce DHPG-LTD (Ronesi and Huber, 2008). In the present study, no significant differences were observed in Homer1 or Homer1b/c protein expression between control and JDM rats (Fig. 2).

Furthermore, DHPG-LTD is considered to involve presynaptic mechanisms, as evidenced by an increase in the PPF ratio during DHPG-LTD (Faas et al., 2002, Kumar and Foster, 2007, Mannaioni et al., 2001, Tan et al., 2003). We confirmed this increase under the high PPF condition (ISIs of 50 ms) after the application of DHPG in control and JDM rats. Since PPF is primarily associated with enhanced presynaptic transmitter release (Zucker, 1989), low PPF is generally interpreted as reflecting a high probability of transmitter release (Hessler et al., 1993, Murthy et al., 1997). In the present study, the low PPF condition (ISIs at 200 ms) was affected by the application of DHPG in JDM rats, but not in control rats (Fig. 3). GABAergic interneurons in the hippocampal CA1 region mediate both feedforward and feedback inhibition of glutamatergic CA1 pyramidal neurons. Rohde et al. (2009) reported that there was no significant effect of a selective GABA_A_ receptor agonist on DHPG-LTD. Although activation of GABA_B_ autoreceptors in the hippocampal CA1 region showed to contribute PPF (Nathan et al., 1990*)*, GABA_B1_ knockout mice exhibited PPF at interstimulus intervals (ISIs) of 10–200 ms that did not differ significantly from that of wild-type mice (Jurado-Parras et al., 2016). Therefore, it remains unclear whether GABA_A_ or GABA_B_ receptors may be involved in DHPG-induced PPF changes in JDM rats.

Another Gq-coupled LTD-dependent mechanism, mAChR-LTD, at CA3-CA1 synapses occurred in both control and JDM rats (Fig. 4). The pharmacological activation of group I mGluRs or M1 mAChRs was found to elicit protein synthesis-dependent LTD, and these forms of LTD occluded each other (Huber et al., 2001, Volk et al., 2007). However, in the present study, mAChR-LTD occurred in JDM rats, whereas mGluR-LTD was attenuated. Group I mGluRs have been shown to modulate glutamate release in different synapse types. Presynaptic mGluRs control intraterminal enzymatic pathways, dictating the migration and fusion of vesicles to the synaptic membranes, and then tuning transmitter outflow (Olivero et al., 2020). Additionally, DHPG-induced increases in neuronal excitability in the hippocampus were mainly attributed to the activation of mGluR5 autoreceptors (Mannaioni et al., 1999). Future studies are needed to investigate the mechanisms by which mGluR5 plays a role in LTD, learning, and memory formation in JDM rats.

We previously demonstrated that electrically induced synaptic plasticity differed depending on age at disease onset (Sasaki-Hamada et al., 2012). We recently examined pharmacologically induced synaptic plasticity and found that mAChR-LTD and mGluR-LTD were both attenuated in young adult-onset STZ rats (Otsuka et al., 2024, Otsuka et al., 2026). In the present study, we investigated juvenile-onset STZ rats and demonstrated that mGluR-LTD was attenuated, whereas mAChR-LTD was not affected. The results obtained herein provide novel insights into neurological complications associated with age at disease onset.

At present, there is no animal model that can fully replicate the condition of human type 1 diabetes. The use of STZ-induced diabetic animal model is a limitation of this study, since some experiments have shown that STZ causes an increased incidence of insulinomas as well as kidney and liver tumors (Singh et al., 2024). Therefore, we will continue our research efforts to improve animal models.

## Author contributions

J-IO and HI conceived and supervised the study; SS-H designed the experiments; SS-H and HO performed the experiments and analyzed data; SS-H and J-IO wrote the manuscript.

## CRediT authorship contribution statement

**Ishibashi Hitoshoi:** Writing – review & editing, Supervision, Funding acquisition. **Jun-Ichiro Oka:** Writing – original draft, Supervision, Funding acquisition. **Hayuma Otsuka:** Writing – review & editing, Formal analysis, Data curation. **Sachie Sasaki-Hamada:** Writing – original draft, Formal analysis, Data curation, Conceptualization.

## Declaration of Generative AI and AI-assisted technologies in the writing process

The authors declare that generative AI was not used in the creation of this manuscript.

## Declaration of Competing Interest

The authors declare that they have no known competing financial interests or personal relationships that could have appeared to influence the work reported in this paper.

## References

[bib1] Balakrishnan S., PK T., Paulose C.S. (2009). Glutamate (mGluR-5) gene expression in brain regions of streptozotocin induced diabetic rats as a function of age: role in regulation of calcium release from the pancreatic islets in vitro. Biomed. Sci..

[bib2] Berthele A., Laurie D.J., Platzer S., Zieglgänsberger W., Tölle T.R., Sommer B. (1998). Differential expression of rat and human type 1 metabotropic glutamate receptor splice variant messenger RNAs. Neuroscience.

[bib3] Bodzęta A., Scheefhals N., MacGillavry H.D. (2021). Membrane trafficking and positioning of mGluRs at presynaptic and postsynaptic sites of excitatory synapses. Neuropharmacology.

[bib4] Bordi F., Ugolini A. (1999). Group I metabotropic glutamate receptors: implications for brain diseases. Prog. Neurobiol..

[bib5] Brakeman P.R., Lanahan A.A., O'Brien R., Roche K., Barnes C.A., Huganir R.L., Worley P.F. (1997). Homer: a protein that selectively binds metabotropic glutamate receptors. Nature.

[bib6] Caulfield M.P., Birdsall N.J. (1998). International Union of Pharmacology. XVII. Classification of muscarinic acetylcholine receptors. Pharmacol. Rev..

[bib7] Collingridge G.L., Peineau S., Howland J.G., Wang Y.T. (2010). Long-term depression in the CNS. Nat. Rev. Neurosci..

[bib8] Dickinson B.A., Jo J., Seok H., Son G.H., Whitcomb D.J., Davies C.H., Sheng M., Collingridge G.L., Cho K. (2009). A novel mechanism of hippocampal LTD involving muscarinic receptor-triggered interactions between AMPARs, GRIP and liprin-alpha. Mol. Brain..

[bib9] Diering G.H., Huganir R.L. (2018). The AMPA receptor code of synaptic plasticity. Neuron.

[bib10] Faas G.C., Adwanikar H., Gereau I.V.R.W., Saggau P. (2002). Modulation of presynaptic calcium transients by metabotropic glutamate receptor activation: a differential role in acute depression of synaptic transmission and long-term depression.. J. Neurosci..

[bib11] Ferguson S.C., Blane A., Wardlaw J., Frier B.M., Perros P., McCrimmon R.J., Deary I.J. (2005). Influence of an early-onset age of type 1 diabetes on cerebral structure and cognitive function. Diabetes Care.

[bib12] Fitzjohn S.M., Irving A.J., Palmer M.J., Harvey J., Lodge D., Collingridge G.L. (1996). Activation of group I mGluRs potentiates NMDA responses in rat hippocampal slices. Neurosci. Lett..

[bib13] Fitzjohn S.M., Kingston A.E., Lodge D., Collingridge G.L. (1999). DHPG-induced LTD in area CA1 of juvenile rat hippocampus; characterisation and sensitivity to novel mGlu receptor antagonists. Neuropharmacology.

[bib14] Geddes J., Deary I.J., Frier B.M. (2008). Effects of acute insulin-induced hypoglycaemia on psychomotor function: people with type 1 diabetes are less affected than non-diabetic adults. Diabetologia.

[bib15] Gimse K., Gorzek R.C., Olin A., Osting S., Burger C. (2018). Hippocampal Homer1b/c is necessary for contextual fear conditioning and group I metabotropic glutamate receptor mediated long-term depression. Neurobiol. Learn. Mem..

[bib16] Hasselmo M.E. (1999). Neuromodulation: acetylcholine and memory consolidation. Trends Cogn. Sci..

[bib17] Hessler N.A., Shirke A.M., Malinow R. (1993). The probability of transmitter release at a mammalian central synapse. Nature.

[bib18] Hormazábal-Aguayo I., Ezzatvar Y., Huerta-Uribe N., Ramírez-Vélez R., Izquierdo M., García-Hermoso A. (2024). Incidence of type 1 diabetes mellitus in children and adolescents under 20 years of age across 55 countries from 2000 to 2022: a systematic review with meta-analysis. Diabetes Metab. Res. Rev..

[bib19] Hou L., Antion M.D., Hu D., Spencer C.M., Paylor R., Klann E. (2006). Dynamic translational and proteasomal regulation of fragile X mental retardation protein controls mGluR-dependent long-term depression. Neuron.

[bib20] Huang C.C., Hsu K.S. (2006). Sustained activation of metabotropic glutamate receptor 5 and protein tyrosine phosphatases mediate the expression of (S)-3,5-dihydroxyphenylglycine-induced long-term depression in the hippocampal CA1 region. J. Neurochem..

[bib21] Huber K.M., Kayser M.S., Bear M.F. (2000). Role for rapid dendritic protein synthesis in hippocampal mGluR-dependent long-term depression. Science.

[bib22] Huber K.M., Roder J.C., Bear M.F. (2001). Chemical induction of mGluR5- and protein synthesis-dependent long-term depression in hippocampal area CA1.. J. Neurophysiol..

[bib23] International Diabetes Federation (2025). IDF Diabetes Atlas.

[bib24] Iwai T., Suzuki M., Kobayashi K., Mori K., Mogi Y., Oka J.-I. (2009). The influences of juvenile diabetes on memory and hippocampal plasticity in rats: improving effects of glucagon-like peptide-1. Neurosci. Res..

[bib25] Jurado-Parras M.T., Delgado-García J.M., Sánchez-Campusano R., Gassmann M., Bettler B., Gruart A. (2016). Presynaptic GABAB Receptors Regulate Hippocampal Synapses during Associative Learning in Behaving Mice. PLoS. One.

[bib26] Kalinowska M., Francesconi A. (2016). Group I metabotropic glutamate receptor interacting proteins: fine-tuning receptor functions in health and disease. Curr. Neuropharmacol..

[bib27] Kammermeier P.J. (2008). Endogenous homer proteins regulate metabotropic glutamate receptor signaling in neurons. J. Neurosci..

[bib28] Katz B., Miledi R. (1968). The role of calcium in neuromuscular facilitation. J. Physiol..

[bib29] Kumar A., Foster T.C. (2007). Shift in induction mechanisms underlies an age-dependent increase in DHPG-induced synaptic depression at CA3-CA1 synapses. J. Neurophysiol..

[bib30] Luján R., Nusser Z., Roberts J.D.B., Shigemoto R., Somogyi P. (1996). Perisynaptic location of metabotropic glutamate receptors mGluR1 and mGluR5 on dendrites and dendritic spines in the rat hippocampus. Eur. J. Neurosci..

[bib31] Malenka R.C., Bear M.F. (2004). LTP and LTD: an embarrassment of riches. Neuron.

[bib32] Mannaioni G., Attucci S., Missanelli A., Pellicciari R., Corradetti R., Moroni F. (1999). Biochemical and electrophysiological studies on (S)-(+)-2-(3′-carboxybicyclo(1.1.1)pentyl)-glycine (CBPG), a novel mGlu5 receptor agonist endowed with mGlu1 receptor antagonist activity. Neuropharmacology.

[bib33] Mannaioni G., Marino M.J., Valenti O., Traynelis S.F., Conn P.J. (2001). Metabotropic glutamate receptors 1 and 5 differentially regulate CA1 pyramidal cell function. J. Neurosci..

[bib34] McAulay V., Deary I.J., Sommerfield A.J., Matthews G., Frier B.M. (2006). Effects of acute hypoglycemia on motivation and cognitive interference in people with type 1 diabetes. J. Clin. Psychopharmacol..

[bib35] Murthy V.N., Sejnowski T.J., Stevens C.F. (1997). Heterogeneous release properties of visualized individual hippocampal synapses. Neuron.

[bib36] Nathan T., Jensen M.S., Lambert J.D. (1990). GABAB receptors play a major role in paired-pulse facilitation in area CA1 of the rat hippocampus.. Brain Res..

[bib37] Oliet S.H., Malenka R.C., Nicoll R.A. (1997). Two distinct forms of long-term depression coexist in CA1 hippocampal pyramidal cells. Neuron.

[bib38] Olivero G., Vergassola M., Cisani F., Roggeri A., Pittaluga A. (2020). Presynaptic release-regulating metabotropic glutamate receptors: an update. Curr. Neuropharmacol..

[bib39] Otsuka H., Sasaki-Hamada S., Ishibashi H., Oka J.-I. (2024). Hippocampal acetylcholine receptor activation-dependent long-term depression in streptozotocin-induced diabetic rats. Neurosci. Lett..

[bib40] Otsuka H., Sasaki-Hamada S., Ishibashi H., Oka J.-I. (2026). Attenuation of mGluR1/5-dependent synaptic plasticity and ERK pathway dysfunction in the hippocampus of diabetic rats. Front. Neurosci..

[bib41] Palmer M.J., Irving A.J., Seabrook G.R., Jane D.E., Collingridge G.L. (1997). The group I mGlu receptor agonist DHPG induces a novel form of LTD in the CA1 region of the hippocampus. Neuropharmacology.

[bib42] Rohde M., Tokay T., Köhling R., Kirschstein T. (2009). GABA_A_ receptor inhibition does not affect mGluR-dependent LTD at hippocampal Schaffer collateral-CA1 synapses.. Neurosci. Lett..

[bib43] Ronesi J.A., Huber K.M. (2008). Homer interactions are necessary for metabotropic glutamate receptor-induced long-term depression and translational activation.. J. Neurosci..

[bib44] Sacai H., Sasaki-Hamada S., Sugiyama A., Saitoh A., Mori K., Yamada M., Oka J.I. (2014). The impairment in spatial learning and hippocampal LTD induced through the PKA pathway in juvenile-onset diabetes rats are rescued by modulating NMDA receptor function. Neurosci. Res..

[bib45] Sasaki-Hamada S., Sacai H., Oka J.-I. (2012). Diabetes onset influences hippocampal synaptic plasticity in streptozotocin-treated rats. Neuroscience.

[bib46] Scheiderer C.L., McCutchen E., Thacker E.E., Kolasa K., Ward M.K., Parsons D., Harrell L.E., Dobrunz L.E., McMahon L.L. (2006). Sympathetic sprouting drives hippocampal cholinergic reinnervation that prevents loss of a muscarinic receptor-dependent long-term depression at CA3-CA1 synapses. J. Neurosci..

[bib47] Singh R., Gholipourmalekabadi M., Shafikhani S.H. (2024). Animal models for type 1 and type 2 diabetes: advantages and limitations.. Front. Endocrinol. (Lausanne).

[bib48] Tan Y., Hori N., Carpenter D.O. (2003). The mechanism of presynaptic long-term depression mediated by group I metabotropic glutamate receptors. Cell. Mol. Neurobiol..

[bib49] Tu J.C., Xiao B., Yuan J.P., Lanahan A.A., Leoffert K., Li M., Linden D.J., Worley P.F. (1998). Homer binds a novel proline-rich motif and links group 1 metabotropic glutamate receptors with IP3 receptors. Neuron.

[bib50] Volk L.J., Pfeiffer B.E., Gibson J.R., Huber K.M. (2007). Multiple Gq-coupled receptors converge on a common protein synthesis-dependent long-term depression that is affected in fragile X syndrome mental retardation. J. Neurosci..

[bib51] Warburton E.C., Koder T., Cho K., Massey P.V., Duguid G., Barker G.R., Aggleton J.P., Bashir Z.I., Brown M.W. (2003). Cholinergic neurotransmission is essential for perirhinal cortical plasticity and recognition memory. Neuron.

[bib52] Zucker R.S. (1989). Short-term synaptic plasticity. Annu. Rev. Neurosci..

